# GPR6 Structural Insights: Homology Model Construction and Docking Studies

**DOI:** 10.3390/molecules25030725

**Published:** 2020-02-07

**Authors:** Israa H. Isawi, Paula Morales, Noori Sotudeh, Dow P. Hurst, Diane L. Lynch, Patricia H. Reggio

**Affiliations:** 1 Department of Chemistry and Biochemistry, University of North Carolina at Greensboro, Greensboro, NC 27412, USA; ihisawi@uncg.edu (I.H.I.); dphurst@uncg.edu (D.P.H.); dllynch@uncg.edu (D.L.L.); 2 Instituto de Química Medica (IQM-CSIC), C/Juan de la Cierva 3, 28006 Madrid, Spain; paula.morales@iqm.csic.es; 3 Department of Physiology and Biophysics, The State University of New York at Buffalo, Buffalo, NY 14260, USA; nouroddi@buffalo.edu

**Keywords:** GPR6, homology model, molecular modeling, G-protein-coupled receptor, molecular dynamics, molecular docking

## Abstract

GPR6 is an orphan G protein-coupled receptor that has been associated with the cannabinoid family because of its recognition of a sub-set of cannabinoid ligands. The high abundance of GPR6 in the central nervous system, along with high constitutive activity and a link to several neurodegenerative diseases make GPR6 a promising biological target. In fact, diverse research groups have demonstrated that GPR6 represents a possible target for the treatment of neurodegenerative disorders such as Parkinson’s disease, Alzheimer’s disease, and Huntington’s disease. Several patents have claimed the use of a wide range of pyrazine derivatives as GPR6 inverse agonists for the treatment of Parkinson’s disease symptoms and other dyskinesia syndromes. However, the full pharmacological importance of GPR6 has not yet been fully explored due to the lack of high potency, readily available ligands targeting GPR6. The long-term goal of the present study is to develop such ligands. In this paper, we describe our initial steps towards this goal. A human GPR6 homology model was constructed using a suite of computational techniques. This model permitted the identification of unique GPR6 structural features and the exploration of the GPR6 binding crevice. A subset of patented pyrazine analogs were docked in the resultant GPR6 inactive state model to validate the model, rationalize the structure-activity relationships from the reported patents and identify the key residues in the binding crevice for ligand recognition. We will take this structural knowledge into the next phase of GPR6 project, in which scaffold hopping will be used to design new GPR6 ligands.

## 1. Introduction

G-protein-coupled receptors (GPCRs) are one of the biggest therapeutic targets, as they comprise around 30% of all current drug targets [1]. GPCRs are involved in a myriad of physiological processes and ailments, including neurodegenerative disorders [2]. GPR6 is a 362 amino acid, rhodopsin-like Class A GPCR orphan receptor, that was cloned in 1995 [3,4]. Phylogenetically, GPR6 belongs to the MECA (Melanocortin/Endothelial differentiation/Cannabinoid/Adenosine) cluster [5,6]. Both sphingosine-1- phosphate (S1P) and sphingosylphosphorylcholines (SPC) have been suggested to be GPR6 endogenous ligands [7,8]. However, due to contradictory reports, the International Union of Basic and Clinical Pharmacology (IUPHAR) still considers GPR6 to be an orphan receptor [9]. Very recently endocannabinoid-like N-acylamides such as N-arachidonoyl dopamine, N-oleoyl dopamine and N-palmitoyl dopamine have been shown to exert inverse agonism at GPR6 in the micromolar range [10]. Some phytocannabinoids such as cannabidiol [11], and cannabinoid antagonists including SR144528 [12], and aminoalkylindole cannabinoid agonist WIN55212-2 [12,13] have shown activity on GPR6.

GPR6 is a Gαs-coupled receptor that its highly expressed in human striatum and hypothalamus [14]. It exhibits high constitutive activation of adenylyl cyclase, producing cyclic AMP (cAMP) levels that rival the cAMP levels produced by many fully activated Gαs-coupled receptors with their corresponding agonists [4,7,15].

GPR6 has been suggested as a possible target for the treatment of Parkinson’s disease [16,17]. Experimental evidence shows that depletion of GPR6 causes both dopamine increase and cAMP decrease in the striatal tissues which are accompanied by improved movement [16]. Knocking out GPR6 in a Parkinson’s disease mice model results in a decrease of the involuntary movements that characterize this disease. Diminishing the constitutive signaling of GPR6 is currently being pursued by the pharmaceutical industry as a therapeutic approach for the management of Parkinson’s disease [18,19,20,21,22]. In fact, several patents claim the use of pyrazine derivatives as GPR6 inverse agonists for treating Parkinson’s disease symptoms and other dyskinesia syndromes [18,19,20,21,22]. Some of these analogs showed a nanomolar activity in cAMP accumulation assays in *h*GPR6-CHO cells. Using a Haloperidol induced Parkinson’s disease rodent model, some ligands showed the capability of reversing catalepsy in a dose-dependent manner. GPR6 is involved in other neurological disorders such as Huntington’s [23,24] and Alzheimer’s Disease [25]. Moreover, instrumental learning has been shown to be impaired in GPR6 knockout mice models [26]. In 2001, Arena Pharmaceuticals. Inc, filed a patent application regarding the use of imidazolidinethiones and imidazodithiazoles derivatives as GPR6 inverse agonists for treating clinical obesity [27].

In contrast to the growing therapeutic interest, little is known about this orphan receptor. The scarcity of identified ligands along with the lack of GPR6 structural information, is delaying therapeutic exploitation. No GPR6 X-ray crystal, cryo-electron microscopy, or nuclear magnetic resonance (NMR) structural studies have been published. Therefore, we have developed a detailed structural study of GPR6, including the construction of the first complete set of GPR6 molecular models. These models will provide not only the structure of orthosteric binding site of GPR6 but also the membrane bilayer environment of GPR6. This computational model was used to reveal GPR6 structural features; to explore the binding crevice; to rationalize the structure-activity relationships from the reported patents; to identify the critical residues in the binding crevice for ligand recognition; and, to guide future drug design.

## 2. Results and Discussion

### 2.1. GPR6 Inactive-State Model Development

The *h*GPR6 homology model was constructed based upon the X-ray crystal structure of the Sphingosine-1-phosphate receptor 1 (S1P1) at a 2.8 Å resolution [PDB identifier: 3V2Y] [28]. S1P1 shares 33% sequence homology (similarity) with GPR6 sequence [14]. Both share essential features such as (1) the absence of helix kinking proline residues in TMH2 (2.58 or 2.59) and TMH5 (5.50), (2) the presence of an acidic residue E1.49 before the highly conserved N1.50 in TMH1 and (3) the presence of an internal disulfide bridge in the EC2 loop (see Supplementary Materials, Figure S1 for Human sequence alignments of S1P1 and GPR6 receptors). Further details regarding template selection are discussed in the Methods Section 3.2. After mutating the S1P1 sequence to the GPR6 sequence, we identified the presence of additional helix bending residues in GPR6 that were not present in S1P1. These occur in TMH1 (T1.44), TMH6 (T6.43) and TMH7 (P7.41) (see residues in red in Helix Net below). The Conformational Memories (CM) technique was used to explore the impact of each of these residues on the geometry of these TMHs. CM provides a set of low-free energy conformations by using multiple Monte Carlo/simulated annealing random walks employing the CHARMM (Chemistry at HARvard Molecular Mechanics) force field as described in the Methods Section 3.3 [29,30].

#### 2.1.1. TMH1

**Figure 1:**
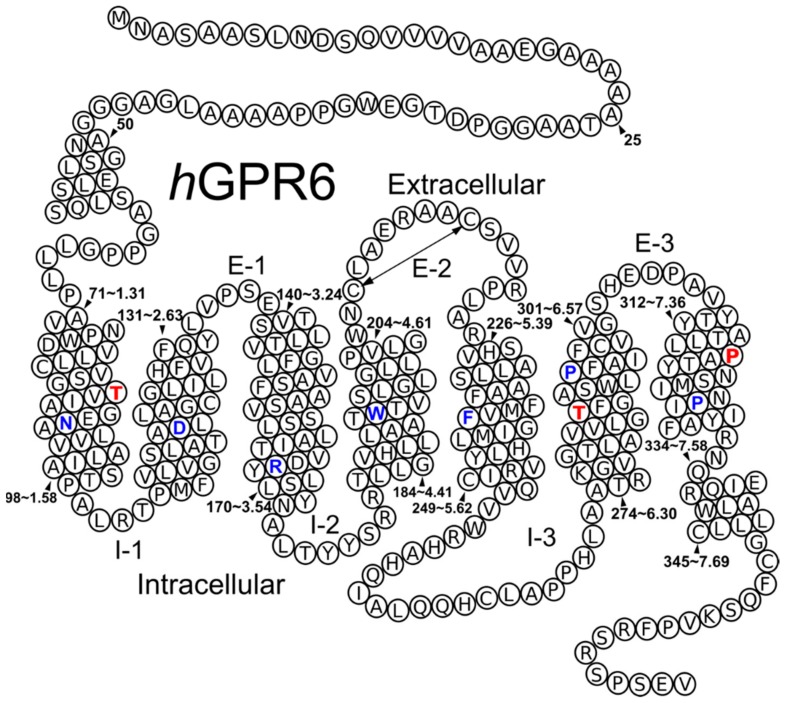
Helix net representation of the human GPR6 sequence. The most highly conserved residue in each helix is colored blue. A key disulfide bridge between the extracellular loop 2 (EC-2) is indicated by a double headed arrow. Black arrowheads indicate specific residue numbers in the GPR6 sequence (absolute and Ballesteros–Weinstein numbering). The additional helix bending residues in TMH1, TMH6 and TMH7 are that were colored red.

In TMH1 of GPR6, there is a threonine that is not present in the S1P1 sequence (see Supplementary Materials, Figure S1). Threonine can bend alpha-helices when its χ1 dihedral is in a g- conformation. Typically, this is seen when a TMH threonine faces the lipid bilayer [31,32]. This is due to their ability to induce an intrahelical hydrogen bond between the side-chain oxygen atom of the threonine and the i-3 or i-4 carbonyl oxygen of the helix backbone. In GPR6, this residue, T1.44, faces lipid and is embedded in an SGT motif. G1.43 can add more flexibility to the region increasing helix bending (Figure 1) [33,34,35]. CM was used to investigate the “hinge region” created by the helix bending residue and the four residues that precede it (T1.44 to C1.40). After superimposing the 112 generated conformers on V1.45 to T1.60, the intracellular amino acids of TMH1 preceding the explored hinge region, most helices were bent away from the binding crevice or toward TMH2 or TMH7. This proves the ability of the SGT motif to bend TMH1. However, there were output helices that would fit in the TMH bundle. The helix conformer selected for incorporation into the GPR6 model was chosen because it forms good Van der Waals interactions with the juxtaposed TMH2 and TMH7 avoiding gaps between them. For helix geometry details, see Table 1.

#### 2.1.2. TMH6

A very striking feature was identified in GPR6 TMH6 sequence. This helix contains an unusually large number of helix bending residues. There are five glycine residues (G6.33, G6.35, G6.42, G6.45 and G6.58), a threonine at T6.43, as well as the conserved proline at P6.50. Many of these residues are not shared with the S1P1 sequence (see Supplementary Materials, Figure S1). These include glycine at positions 6.45, and 6.42 and threonine at position 6.43 that is facing lipid. The region containing these unique residues (L6.41 to G6.45), immediately preceding the conserved SWXP motif (CWXP in most Class A GPCRs), was explored using CM. TMH6 undergoes a conformational transition from an inactive (R) to a G-protein activated state (R*) conformation as part of the GPCR signaling mechanism. This gives exploration of accessible conformations of TMH6 in this region, added importance. This will be described in further detail in Section 2.1.6.

The resultant CM calculated helices were superimposed at their extracellular ends with the backbone residues from A6.46 to V6.57 in the template. The conformers that were generated by CM that had the χ1 of T6.43 in g- were more kicked away from the bundle compared to the conformers with the T6.43 χ1 in g+. The latter set was used to select the conformer that was used in the inactive state bundle. Conformer choice was predicated on its ability to preserve the R3.50-T6.30 ionic lock interaction that characterizes the GPR6 inactive state. In addition, the selected low-free energy conformer fits in the bundle avoiding Van der Waals overlaps with the nearby residues from the other helices. See Table 1 for helix geometry details.

#### 2.1.3. TMH7

The highly conserved NPXXY motif in TMH7 is present in both S1P1 and GPR6. However, GPR6 has an additional proline at position P7.41 that is not present in S1P1 (see Supplementary Materials, Figure S1). As proline residues are known to introduce a kink between the segments preceding and following the proline residue [37,38,39], the presence of P7.41 may bend the top of TMH7 which can affect the overall shape of the binding pocket. To explore its effect on helix shape, P7.41 and four residues prior to it (A7.37 to P7.41) backbone dihedrals were varied using CM. The values used for range variations according to the Protein Data Bank were: −120° < ϕ < 40°, −70° < ψ < 0° and −160° < ω < 160° [40]. The 112 conformers generated were superimposed from P7.41 to R7.56, the intracellular amino acids of TMH7 preceding the explored hinge region with the varied proline. CM calculations suggest that the extracellular end of TMH7 could be tilted inwards, occluding the binding site. However, the selected TMH7 conformer does not invade the binding crevice or generate major steric overlap with other helices of the GPR6 bundle. See Table 1 for helix geometry details.

#### 2.1.4. N-Terminus

The N-terminus (N-term) of GPR6 is composed of 70 amino acids, which is long compared to other Class A GPCRs. Cannabinoid receptor type 1 (CB1) is the only GPCR that has a longer N-term than GPR6 in the MECA cluster [5,6]. It has 112 residues in N-term. Prime Homology modeling implemented in the Schrödinger package was used to model the last 25 residues of the GPR6 N-term that are adjacent to TMH1. According to crystal structures of similar lipid sensing proteins as S1P1 and LPA1, the N-term may contain a helical segment [28,41]. The following structural features were taken into consideration when modeling and selecting the GPR6 N-term. These include a helical N-term region between G(52)–S(61), because its sequence presents a periodicity between polar and nonpolar residues, which suggest an alpha helical segment, and contains NGS motif in there (NXS/T is an N-glycosylation site) [42] that should be exposed to extracellular milieu, the ability to fit with the other EC loops forming hydrophobic patches with them, and the ability to block access of ligands from the extracellular milieu [43], while diminishing water access to the binding site.

#### 2.1.5. EC2

Important structural features were taken into account in modeling GPR6 loops using MODELLER 8.2 (copyright © 2020–2017 Andrej Sali, San Francisco, CA, USA). In GPR6, the EC2 displays the CX_6_CX_4_P motif that is present in other receptors, indicating that a limited distance should be introduced between C(209) and C(216) to form a conserved internal disulfide bridge in the MECA cluster (Figure 1) [28,41]. As in the crystal structures of S1P1 and LPA1, the third and the fourth residues after the last cysteine in the disulfide bridge points down, invading the binding crevice. These two residues are V(219) and R(220) in GPR6. The latter shown to have a critical role in the binding crevice as a main Hydrogen Bond Donor (HBD) as will be shown in Section 2.2.3.

#### 2.1.6. Ionic Lock and Toggle Switch

**Figure 2:**
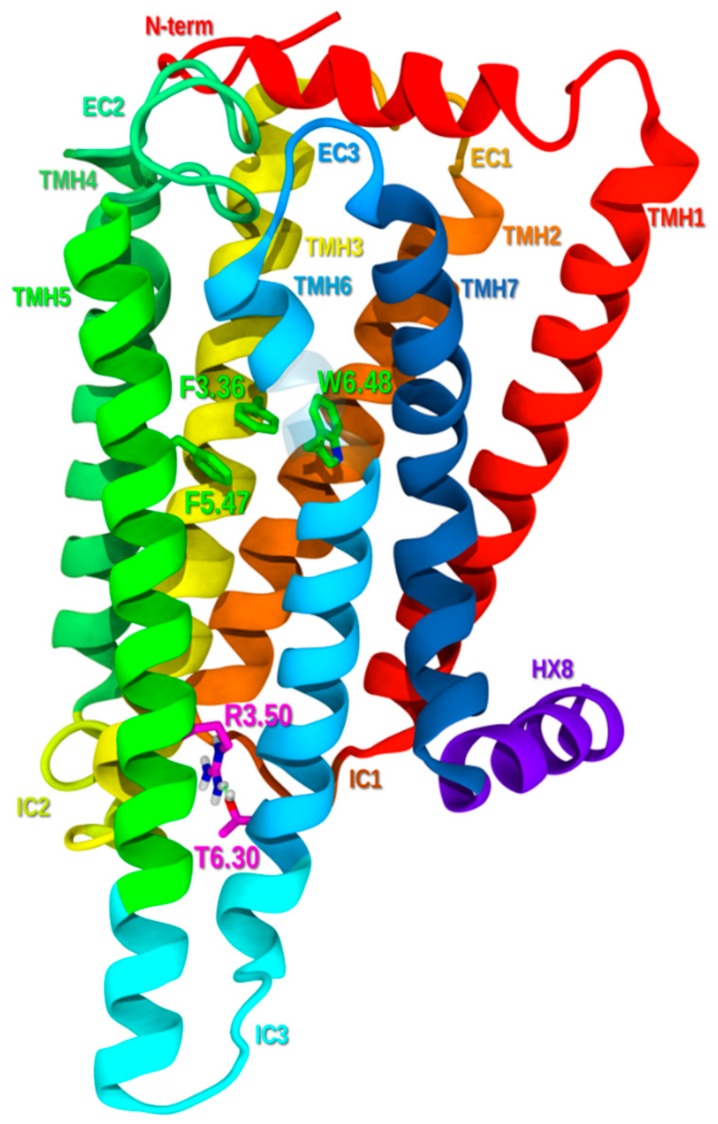
Lipid view of GPR6 from TMH5-TMH6 portal (portion of TMH6 made transparent for better view). Toggle switch residues (F3.36-W6.48-F5.47) are shown in green and ionic lock residues (R3.50-T6.30) are shown in magenta.

GPCRs can signal through a G-protein dependent signaling pathway and/or a G-protein independent signaling pathway via β-arrestin [44,45]. G-protein dependent signaling requires an opening form at the intracellular end of the receptor for the G-protein to complex. While the G-protein independent signaling pathway via β-arrestin requires TMH7 movement to expose the extreme end of the C-terminus for the β-arrestin to couple with it [46]. GPR6 has been shown to signal via G-protein and to have high constitutive activity [14]. Activation via G-protein occurs upon changes in intrahelical interactions between TMH3 and TMH6 [47]. Specifically, the receptor activates when TMH3 undergoes a counter-clockwise rotation, while TMH6 straightens at P6.50 moving its intracellular end away from TMH3, breaking a key salt bridge known as the “ionic lock” (R3.50-T6.30) at the intracellular end of the helix bundle. In GPR6, the “ionic lock” is a good Hydrogen bond formed between R3.50 and T6.30. When this “lock” is formed, the receptor is in its inactive state (R) with no opening on the intracellular side of the TMH bundle (Figure 2). When the “lock” is broken, the IC ends of TMHs 3 and 6 move apart by undergoing helix conformational change to produce the G-protein activated state (R*) [45]. The transition from the R to R* states is usually triggered by agonist entry into the ligand binding pocket which causes the binding pocket “Toggle Switch” to change conformation [48]. In GPR6, this toggle switch is formed by F3.36 and W6.48 (Figure 2). In the inactive state, the chi1(χ1) dihedral angle of W6.48 is in a g+ conformation. W6.48 is held in a g+ chi1 by another residue, F3.36 (χ1 in trans). During activation, ligand binding causes the F3.36 χ1 dihedral angle to change from trans to g+. This releases W6.48 to undergo its χ1 change from g+ to trans. As W6.48 is part of the TMH6 hinge motif, CWXP (SWXP in GPR6), changes in W6.48 can induce a straightening of TMH6. In GPR6, the toggle switch may include a third residue, F5.47, that has an aromatic stacking interaction with W6.48. This residue in the R state is in a trans χ1 rotameric state that changes to a g+ χ1 as GPR6 is activated.

Recently, GPR6 has been shown to also signal through a G-protein independent, β-arrestin signaling pathway. GPR6 displays high constitutive activity in β-arrestin2 recruitment assays in CHO cells co-expressing GPR6-PK1 and EA-β-arrestin2 [11,12]. The IC conformational changes due to the β-arrestin activation mechanism are not fully elucidated and studied as G-protein activation among GPCRs. It has been suggested that conformational changes occur in TMH7-Hx8 domains accompanying β-arrestin signaling [49,50], with no changes in the conformational status of TMH3/TMH6, keeping the ionic lock intact.

#### 2.1.7. Inactive State Model Minimization

The resultant homology model has been optimized using OPLS3e all atom force field (applying an 8.0-Å extended nonbonded cutoff, a 20.0-Å electrostatic cutoff, and a 4.0-Å hydrogen bond cutoff) as described previously in the Reggio lab protocol [51]. The minimized, inactive state GPR6 bundle preserves the following main class A GPCR interhelical contacts: (1) the hydrogen bond networks in TMH1-2-7 (N1.50-D2.50-N7.49), in TMH1-7 (E1.49-X7.47), and, in TMH4-2-3 (W4.50-S2.45-S3.42); (2) aromatic stacking between TMH3-6-5 toggle switch residues (F3.36-W6.48-F5.47); and, (3) the good hydrogen bond between TMH3-6 residues (R3.50-T6.30) as the ionic lock.

**Figure 3:**
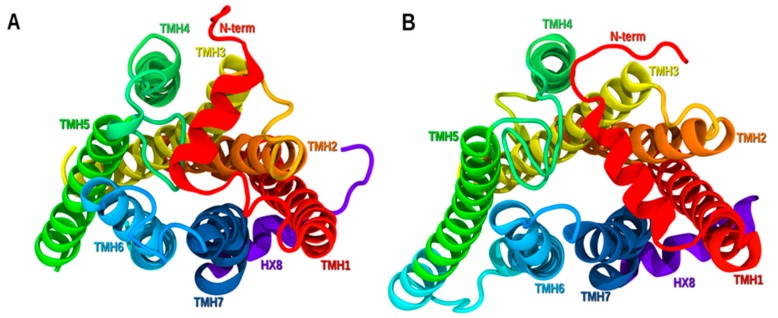
A comparison of (**A**) the S1P1 inactive state crystal structure and (**B**) the GPR6 inactive state model. The view here is from the extracellular side of each bundle.

Figure 3, shows a comparison of the S1P1 crystal structure [28] (Figure 3A) and the GPR6 inactive state model developed here (Figure 3B) from an extracellular view. The main differences seen in TMHs are shown here. The EC portion of TMH1 in GPR6 is pulling away from the binding crevice as a result of the presence of SGT motif. Particularly, T1.44 facing lipid causes that bend as mentioned in Section 2.1.1. The top of TMH6 is similar in both receptors, while GPR6 IC is more kicked toward TMH5, preserving the ionic lock interaction. The top of TMH7 in GPR6 bends more toward the binding crevice due to the effect of P7.41 that is not present in S1P1. The N-term contains a helical portion in both S1P1 and GPR6. As in other lipid receptors, the N-term and EC loops cover the top of the bundle shielding it from the extracellular milieu.

In the next sections, the GPR6 model developed here will be used to explore the binding crevice, to rationalize the structure-activity relationships from the reported patents, and, to identify critical residues in the binding crevice using docking studies.

#### 2.1.8. Molecular Dynamics

**Figure 4:**
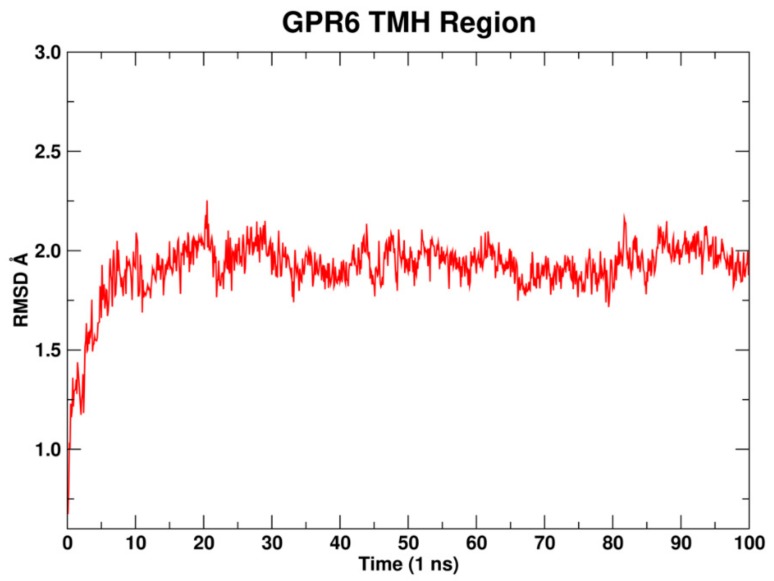
Plot of TMH region root mean square deviation (RMSD) versus simulation time for the GPR6 inactive state model.

Molecular dynamic (MD) simulations of the developed GPR6 inactive state model was performed to validate the stability of the model. GPR6 was embedded in a fully hydrated 1-palmitoyl-2-oleoyl-sn-glycero-3-phosphocholine (POPC) lipid bilayer and simulated for 100 ns. The Orientation of Proteins in Membranes (OPM) was used as a reference for orienting GPR6 [52]. In the Method Section 3.6, full setup details of MD simulations are provided. As seen in Figure 4, the root mean square deviation (RMSD) of the backbone TMHs region is averaged around 2, demonstrating a stabilized bundle through the 100 ns simulation time.

### 2.2. Docking Study and SAR

#### 2.2.1. Structure-Activity Relationship (SAR) Analysis for Pyrazine Analogs

As previously mentioned, very few chemotypes have been identified for GPR6 thus far. Envoy Therapeutics, Inc. and Takeda Pharmaceutical Company developed a large series of pyrazine derivatives as novel GPR6 inverse agonists [18,19,20,21,22]. They claimed the use of these analogs in treating Parkinson’s disease and other dyskinesia syndromes. Five different patents with more than 1000 compounds were reported. Most of them were tested using cAMP accumulation assays in *h*GPR6-CHO cells. In all patents, IC_50_ generated using LANCE^®^ HTRF (homogenous time-resolved fluorescence) Ultra cAMP assay kit with no error bars provided. These series provide structural information that may aid an understanding of important residues in the binding site of GPR6. Our analysis and SAR rationalization for these patented GPR6 modulators is given below.

**Figure 5:**
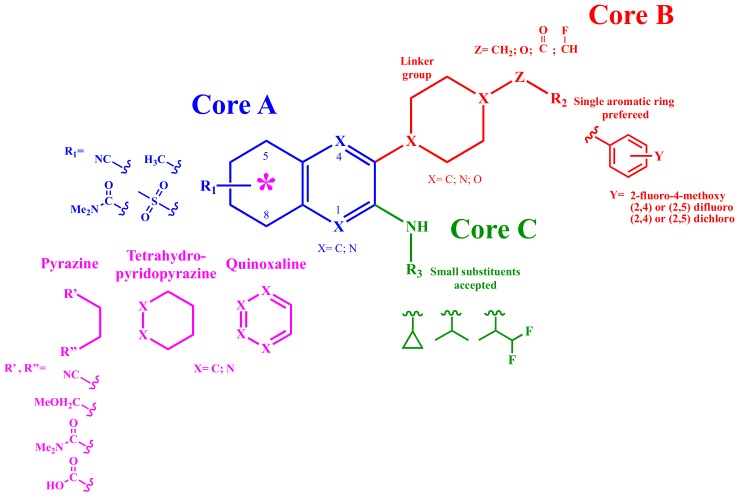
Structure activity relationship (SAR) analysis of Pyrazine analogs. The scheme represents selected examples covered by the patents.

To rationalize the SAR, the structure will be divided into three main cores as depicted in Figure 5. The main structural modifications in the different patents were performed on Core A using moieties of different nature. Initial efforts were directed towards the aromatic bicyclic core A bearing heterobicycles such as quinoxalines, pyridopyrazines or pyrazinopyridazines [18,19]. Many of these compounds exhibited activity in the low nanomolar range. In a second effort, tetrahydropyridopyrazines were explored at Core A showing a decrease in activity to the high micromolar range [20]. Afterwards, a simple heterocycle core A was attempted obtaining nanomolar activity with pyrazine derivatives [21]. In the last patent, a variety of heteroaromatic carboxamides were also investigated [22].

An extensive substitution pattern was explored at R_1_ among all patents. For the bicyclic core, diverse substituents in the 5-, 6- or 7-position were tolerated. However, di-substitution showed decreased activity, especially if position 8 was substituted. On the other hand, compounds with no substituents were tolerated well in terms of activity, as long as there was a nitrogen at position 6. Regarding the pyrazine scaffold, mono- or di- substitution at R’ and R’’ were accepted. For heteroaromatic carboxamides derivatives, substitution with a rich electron density moiety such as a sulfonyl or cyano group at the *meta* or *para* position to the carboxamide was found to be a requirement to maintain high activity.

Core B was explored extensively in the first patent [18] through more than 600 derivatives, which sets out the basis of the structures that were then used in the subsequent patents [19,20,21,22]. The main modifications that were explored are the following:

(1) C_6_ Cycloalkyl or C_6_ heterocycloalkyl as piperidinyl-, piperazinyl-, or morpholino were explored as linkers.
(2) Methylene, oxygen, carbonyl, and flouromethylene were tolerated at the Z position.
(3) Aromaticity is required at R_2_, with a phenyl ring preferred over heterocyclic rings or bicyclic rings. Electron-withdrawing substituents such as fluoro-, chloro- or electron-donating group substituents as methoxy on the aromatic ring are required for higher potency.

For core C, only small substituents such as cyclopropyl-, isopropyl- and difluoroisopropyl- amines conferred good activity, while bulky substituents led to a decrease in the activity.

Analysis of the reported SAR from the patents referenced above revealed the key structural features associated with high activity at GPR6. This allowed selecting a subset of the inverse agonist analogs to be docked in the resultant GPR6 inactive state model, as inverse agonists have higher affinity for the inactive state. Docking studies will be used to validate the model and identify the key residues in the binding crevice for ligand recognition.

#### 2.2.2. Conformational Analysis of Selected GPR6 Inverse Agonists

In docking studies, the global minimum energy conformer is typically docked first. Higher energy conformers are considered if the global minimum does not fit the binding site. This procedure necessitates a previous conformational analysis of subject ligands. Therefore, a systematic conformational analysis for each of the diverse patented inverse agonists was performed using the Spartan 08 molecular modeling program (Wavefunction, Inc., Irvine, CA, USA). Each rotatable bond is rotated either 360° in 120° or 180° increments. The equilibrium geometry for each conformer was calculated by ab initio Hartree-Fock calculations in vacuum at the 6-31G* level. The lowest energy conformer was then used as the initial structure for docking studies.

#### 2.2.3. Docking Studies

**Figure 6:**
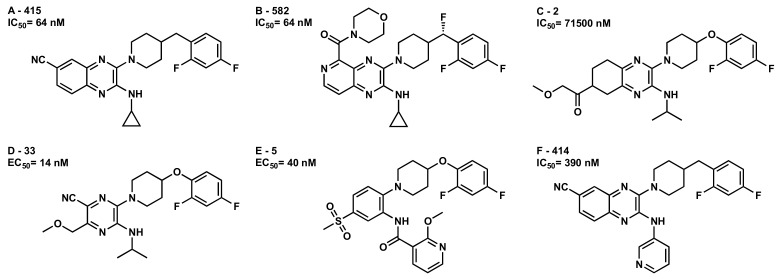
GPR6 modulators—Pyrazine analogs used for docking studies. The numbers next to the alphabetic letter for each compound come from Envoy Therapeutics, Inc. and Takeda Pharmaceutical Company patents.

A selection of representative compounds from the patents was selected to explore the main structural features in our docking studies (Figure 6). At least one from each patent that differs in Core A was chosen to investigate the difference in activities obtained between this series at a molecular level. Those compounds were docked in our inactive state receptor model manually and using the Macromodel Glide algorithm implemented in Schrödinger module [53]. Minimizations of all the complexes were performed in Prime (Schrödinger 2019-3: Prime, Schrödinger, LLC, New York, NY, USA, 2019) using the implicit membrane model, as described in the Methods Section 3.9.

Docking studies showed that these inverse agonists can span the GPR6 orthosteric binding pocket horizontally, suggesting the putative binding site to be in the TMH1-2-3-6-7 region. There is only one positively charged amino acid accessible to ligands in this region, R(220), an EC-2 loop residue. We used this residue as the primary interaction site for studied ligands. The pyrazine analogs can form two main sets of aromatic stacking interactions at the aforementioned TMHs. In the TMH3-6 region, they can hold the rotameric toggle switch, a main switch in the binding pocket that maintains the bundle in the inactive state, by aromatic stacking with F3.36, W6.48 or F6.51. Other aromatic interactions in the TMH1-2-7 region involve residues W1.35, H2.60, F2.61 and/or Y7.36.

**Figure 7:**
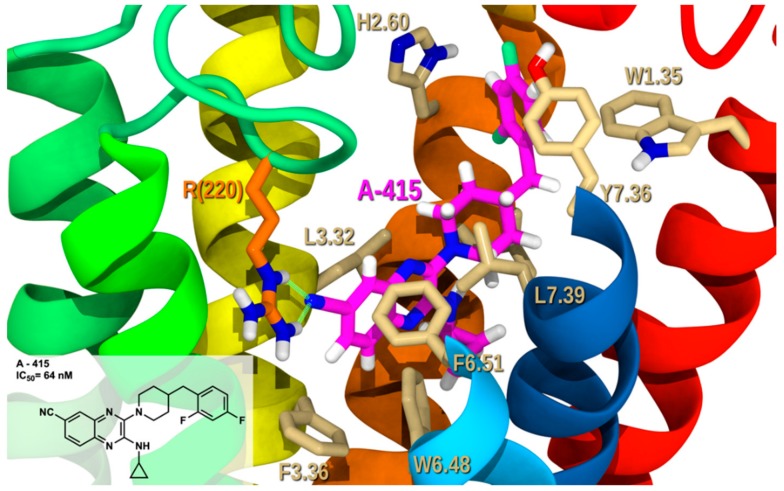
**A-415**/GPR6 R complex. The view from the lipid bilayer with the EC portions of TMH6-7 removed for clarity.

Figure 7 shows analog **A-415** (IC_50_ = 64 nM) in complex with GPR6 R. **A-415** belongs to the aromatic heterobicycle derivatives of core A. The primary interaction is a hydrogen bond formed between the exposed N in the cyano group (the most electronegative site of **A-415**, see Supplementary Materials Figure S2) and EC-2 loop residue, R(200) (both NE and NH2) (see Supplementary Materials Table S1 for interaction measurements). This hydrogen bond helps position the quinoxaline ring between TMH3-6. The quinoxaline ring forms a T-stack with F3.36 and a tilted-T interaction with the 6-membered ring of W6.48. The interhelical aromatic stacking between TMH3-6-5 toggle switch residues (F3.36-W6.48-F5.47) was preserved and stabilized with inverse agonist interaction. On the other side of the receptor, the aromatic ring in core B, the difluorophenyl of **A-415** forms a T-stack with H2.60 and a tilted-T interaction with Y7.36. The 6-membered ring of W1.35 forms an aromatic tilted-T interaction with the difluorophenyl as well. The conformational energy expense for **A-415** is 1.6 kcal/mol. The total pairwise interaction energy for **A-415** at this binding site in GPR6 is −51.71 kcal/mol and the Glide score is −10.10 kcal/mol (see Supplementary Materials Table S2). The major contributions to these interactions come from the hydrogen bonding with R(220), aromatic stacking with W1.35, F3.36, W6.48, and Y7.36 and van der Waals interactions with L(60), L2.57, A2.53, Q(132), L3.32, V3.33, T7.43, F6.51, and L7.39. This docking study shows how aromatic bicyclic derivatives such as quinoxaline analogs, with electron rich substitution at position 6 can fit in GPR6 R, interacting with crucial interaction sites and keeping the bundle in its inactive state.

**Figure 8:**
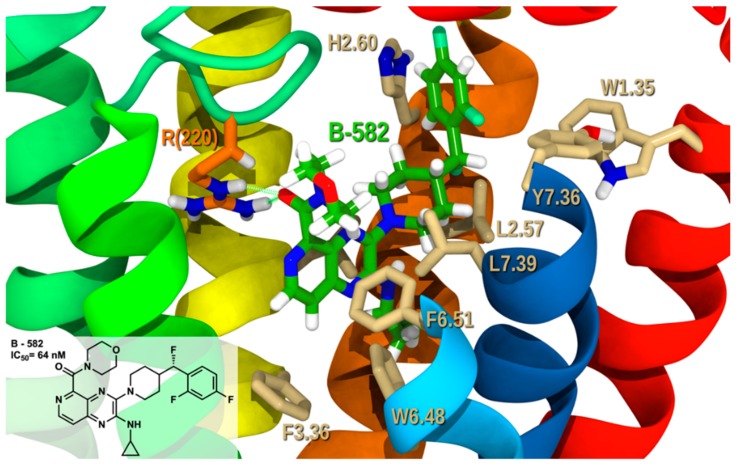
**B-582**/GPR6 R complex. The view from the lipid bilayer with the EC portions of TMH6-7 removed for clarity.

Figure 8 illustrates analog **B-582** (IC_50_ = 64 nM) in complex with GPR6 R. **B-582** belongs to the same patent as **A-415** [18], however **B-582** shows a bioisosteric replacement of a quinoxaline with a pyrido-pyrazine and bears a bulkier substituent at position 5, a morpholino-methanone group. The carbonyl of the morpholino-methanone forms a hydrogen bond with R(220) (see Supplementary Materials Table S1 for interaction measurements). The pyrido ring in pyrido-pyrazine forms a T-stack with both F3.36 and F6.51. While H2.60 forms a T-shaped stacking with the difluorophenyl. The docked conformational cost for **B-582** is 2.5 kcal/mol with total interaction energy of −51.67 kcal/mol with GPR6 R and Glide score of −8.72 kcal/mol (see Supplementary Materials Table S3). The hydrogen bonding with R(220), the aromatic stacking with H2.60, F3.36, and W6.48 and van der Waals interactions with V(219), L2.57, L3.32, V3.33, W6.48, C6.55, L7.39, and T7.43 make a substantial contribution to the total interaction energy. This derivative demonstrates that bulky substituents with exposed rich electron density at position 5 of the bicyclic system are well tolerated by the receptor.

**Figure 9:**
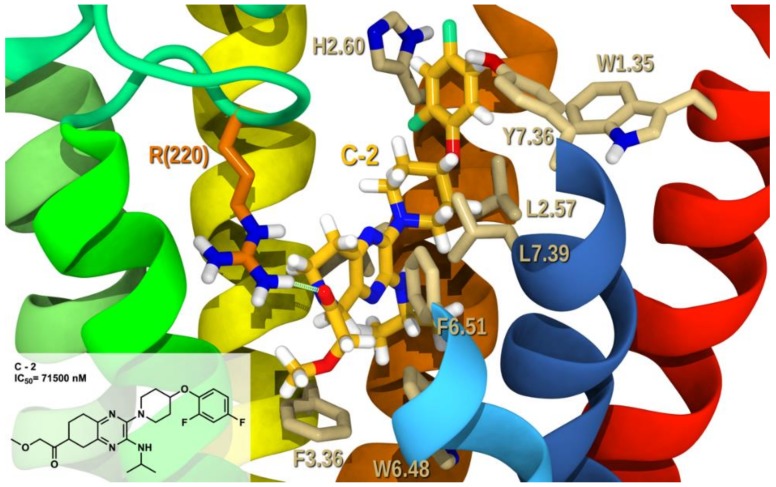
**C-2**/GPR6 R complex. The view from the lipid bilayer with the EC portions of TMH6-7 removed for clarity.

Figure 9 illustrates a tetrahydroquinoxaline analog **C-2** (IC_50_ = 71,500 nM) in complex with GPR6 R. In these derivatives the activity decreased to the micromolar range as a result of losing the aromaticity in the second ring, compared to the previous analogs. Only one hydrogen bond is formed with R(220), as the other hydrogen bond is hindered by the saturated ring hydrogens (see Supplementary Materials Table S1 for interaction measurements). This fused ring is not able to form an aromatic stacking with neither F3.36, nor W6.48, the toggle switch residues. It only formed edge-to-face aromatic stacking with F6.51. The lone pair of the methoxy in the 2-methoxyethanone is pointing to the π cloud core of F3.36, which is a repulsive interaction and further destabilizes the system. The difluorophenyl was still able to form a T-stack with W1.35 and another T-stack with H2.60. The total interaction energy and the conformational cost for such complex are −47.17, and 2.6 kcal/mol, respectively and Glide score of −7.80 kcal/mol (see Supplementary Materials Table S4). The major contribution to this energy is the van der Waals interaction with A2.53, L2.57, V3.29, L3.32, V3.33, F3.36, W6.48 and L7.39, and T7.43. As well, hydrogen bonding to R(220) and aromatic stacking to W1.35, H2.60 and F6.51 contribute to the interaction. The diminished total interaction energy and the lack of aromatic stacking of **C-2** with toggle switch residues cause the reduced ability of **C-2** to act as an inverse agonist, what is in agreement with the reported biological testing.

**Figure 10:**
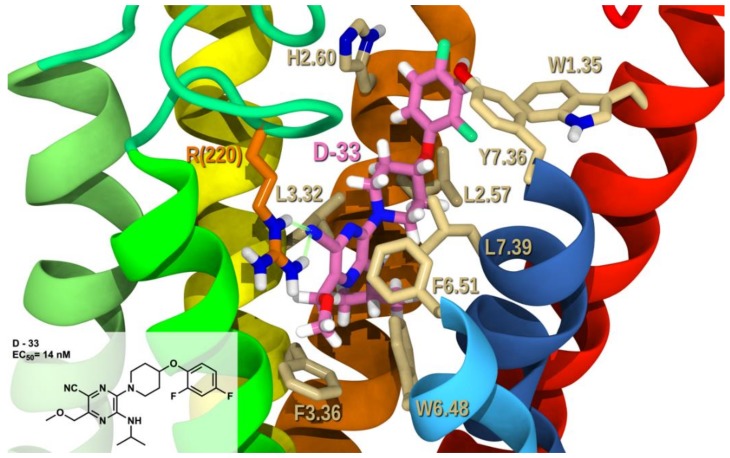
**D-33**/GPR6 R complex. The view from the lipid bilayer with the EC portions of TMH6-7 removed for clarity.

Figure 10 shows derivative **D-33** (EC_50_ = 14 nM) -GPR6 R complex. **D-33** belongs to the monocyclic derivatives of core A. The cyano in **D-33** forms a hydrogen bond with both NE and NH2 of R(220) (see Supplementary Materials Table S1 for interaction measurements). Even with one ring system as core A, the pyrazine is oriented so that it forms aromatic stacking with W6.48 and F6.51. Both W1.35 and H2.60 form T-shaped aromatic stacking with difluorophenyl of **D-33.** The total interaction energy of **D-33** at this docking site in GPR6 is −50.36 kcal/mol with conformational cost of 1.9 kcal/mol and Glide score of −8.71 kcal/mol (see Supplementary Materials Table S5). Hydrogen bonding interactions with R(220) and aromatic stacking with W1.35, H2.60, W6.48 and F6.51, make major contributions to the overall interaction energy. Numerous van der Waals interactions with L2.57, Q(132), L3.32, V3.29, V3.33, F3.36, T7.36, and L7.39 also contribute. This docking study correlates with the biological activity reported, showing the ability of monocyclic derivatives to be good GPR6 inverse agonists, with electron rich substitution at position 5, these can satisfy R(220) and the pyrazine stabilizes toggle switch residues.

**Figure 11:**
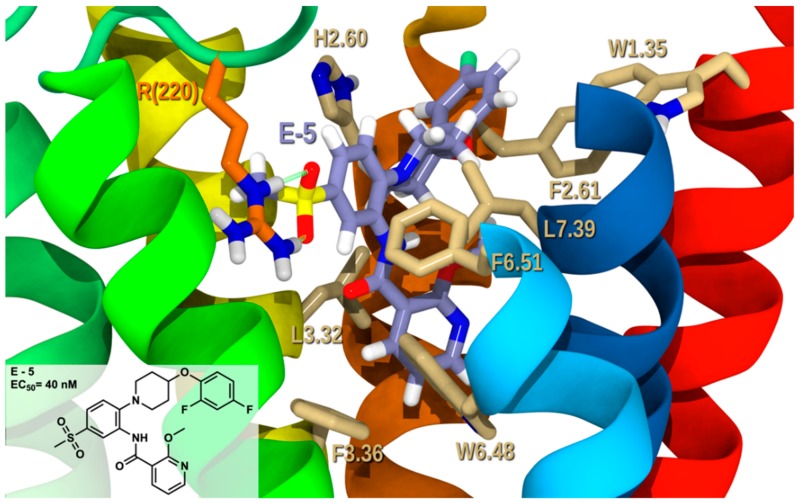
**E-5**/GPR6 R complex. The view from the lipid bilayer with the EC portions of TMH6-7 removed for clarity.

Docking of the heteroaromatic carboxamide derivative **E-5** (EC_50_ = 40 nM) in GPR6 R is illustrated in Figure 11. **E-5** sits slightly differently in the binding crevice compared to the other derivatives due to its structure. Still, this molecule preserves the ability to interact with the crucial receptor sites. In that orientation, the carbonyls in the methylsulfone can form strong hydrogen bonds with R(220) (see Supplementary Materials Table S1 for interaction measurements). In this docking study, we can show the importance of electron rich substitution at *meta* or *para* positions to the heteroaromatic carboxamide, since in that position the electrons are more accessible to a hydrogen bond with R(220). The phenyl in methylsulfonyl-phenyl forms a T-stack with F6.51. While methoxypyridine forms edge-to-face stacking with the 6-membered ring of W6.48. On the other hand, both H2.60 and F2.61 form T-stacks with the difluorophenyl. The conformational cost, the total interaction energy and Glide score for **E-5** at this docking site in GPR6 R is 2.4, −59.22, and −10.38 kcal/mol, respectively (see Supplementary Materials Table S6). The hydrogen bonding interaction with R(220) makes major contributions to the overall interaction. Aromatic stacking interactions with H2.60, F2.61, W6.48, and F6.51 and van der Waals interactions with hydrophobic L2.57, Q(132), V3.29, V3.33, L3.32, L7.39, A7.42, and T7.43 contribute to the overall interaction. This docking study demonstrates that heteroaromatic carboxamide derivatives are good GPR6 inverse agonists, which correlate with the biological activity reported.

The ligand/GPR6 complexes described here can not only rationalize how these molecules bind to GPR6 but also explain structural trends observed in the patents’ SAR. For instance, how bulkier substituents at core C exhibit a decrease in activity. This can be exemplified with analogs **A-415** (IC_50_ = 64 nM) and **F-414** (IC_50_ = 390 nM). Both analogs belong to the aromatic bicyclic core A family. The difference between them lies in core C, where **A-415** bears a small substituent such as cyclopropylamine, and **F-414** bears a bulky one as the aminopyridine. The pyridine ring forces the ligand to sit higher in the binding site, hindering deeper penetration into the crevice to stabilize the toggle switch. Or otherwise pyridine will have edge to edge electrostatic clashes with W6.48 and the lone pairs of pyridine will be clashing with TMH2 residues.

**Figure 12:**
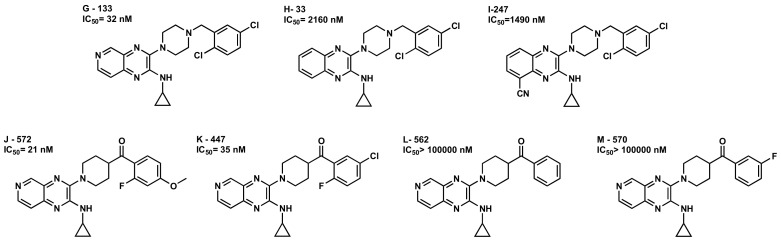
Other GPR6 modulators—Pyrazine analogs. The numbers next to the alphabetic letter for each compound come from Envoy Therapeutics, Inc. and Takeda Pharmaceutical Company patents.

Our docking studies are also consistent with other remarkable structural features reported in the patented series of compounds. The position of the nitrogen or the electron rich substitutions in the aromatic bicyclic core A family play an important role in this family of compounds. As it can be seen in Figure 12 examples, **G-133** (IC_50_ = 32 nM) with the nitrogen at position 6 leads to a good GPR6 inverse agonist. While in **H-33** (IC_50_ = 2160 nM), when the compound loses the nitrogen at position 6, the activity dropped to the micromolar range. Electron rich substitutions at position 8 are not well tolerated, as it can be seen in **I-247** (IC_50_ = 1490 nM) analog. Docking studies of these compounds with their interaction measurements and energies can be seen in the supporting information. In these studies, **G-133** (IC_50_ = 32 nM) the derivative with the nitrogen at position 6 is capable of forming a hydrogen bond with R(220) and a stable aromatic interaction with toggle switch residues (Figure S4/Table S6). While **H-33** (IC_50_ = 2160 nM), is not able to form a hydrogen bond interaction with R(220) (Figure S5/Table S7). In **I-247** (IC_50_ = 1490 nM), the cyano substituent at position 8, provided steric clashes that push the ligand away from forming aromatic interactions with toggle switch residues F3.36 and W6.48, in addition it loses the ability to hydrogen bond with R(220) (Figure S6/Table S8), decreasing its ability to act as a good cAMP inverse agonist.

The 2,4-aromatic substitutions at core B (R_2_) (Figure 5) are preferred over other substitution patterns. This might be due to the fact that they position one edge of the aromatic hydrogens for better interaction at either H2.60 or Y7.36, without the steric hindrance of bulkier substituents as seen in earlier docking studies (Figure 7, Figure 8, Figure 9, Figure 10 and Figure 11). Even though, other substitution patterns are also well accepted such as 2,5, exemplified in comparison between analogs **J-572** (IC_50_ = 21 nM), **K-447** (IC_50_ = 35 nM) and **G-133** (IC_50_ = 32 nM) in Figure 12. As it can be seen in Figure S4, the dichlorophenyl of **G-133** (IC_50_ = 32 nM) was able to form aromatic stacking with Y7.36. While according to the patents, no substitutions, even mono substitution lead to a significant activity decrease as exemplified by analogs **L-562** (IC_50_ > 100,000 nM) and **M-570** (IC_50_ > 100,000 nM) in Figure 12. These changes were explored only in the first patent [18], then most 2.4 and to lesser extent 2.5 substitutions were used for the others [19,20,21,22].

As shown from the present study, GPR6 R can accommodate horizontally oriented inverse agonists to fulfill three main interacting regions for holding the receptor in its inactive state. Fulfilling the positively charged amino acid R(220) from EC2 with a hydrogen bond interaction with an accessible HBA in the inverse agonist ligands. Blocking the toggle switch from moving by aromatic stacking with at least two of F3.36, W6.48 or F6.51. Occupying the aromatic region between TMH1,2 and 7 with aromatic interaction with one or more of W1.35, H2.60, F2.61 and Y7.36, respectively. The hydrophobic residues L2.57, L3.32, and L7.39, that surround the binding crevice at positions shown to be a typical interaction site with ligands in many GPCRs [54,55,56], provided the major van der Waals interactions for the studied molecules in this model. Our docking results can consistently explain at a molecular level the main highlights observed in the analyzed SAR from the patents.

### 2.3. Proposed Mutagenesis for Future Perspectives

In the next phase of this study, we will use site-directed mutagenesis studies to test our homology model and ligand docking studies. The first task will be radiolabeling a high affinity GPR6 ligand. The GPR6 patent literature contains a selection of nanomolar efficacy ligands that could be synthesized and evaluated as radioligands. Once a radioligand is available, there are three regions of contact between the pyrazine analogs and the receptor that will be probed first.

**Site 1: TMH3 and EC-2 Loop.** In the TMH3/EC-2 loop region, docking studies suggest that EC-2 loop residue, R220 is a direct interaction site for pyrazine analogs and provides the highest interaction energy with these analogs. We will generate R220E/M/A mutants to test the importance of this residue. A R220E mutation would show the role of basic vs. acidic residue at this position. Because E220 would be unable to form an interaction with the pyrazine analogs, we expect that this mutation will result in a significant increase in Ki. A R220M mutation removes the potential for ionic or hydrogen bonding but preserves bulk at this position and would create a Van der Waals interaction with many of the ligands. We would still expect an increase in Ki with this mutation, but a smaller increase than the R220E mutation. Finally, an R220A mutation would reduce bulk and provide no ability to form a hydrogen bond. This should also lead to a large increase in Ki.

**Site 2: Toggle Switch Region (TMH3/6).** Core A of the pyrazine analogs forms aromatic stacking interactions with F3.36, F6.51, and W6.48. In Class A GPCRs, W6.48 is the binding pocket residue that flexes the proline kink in TMH6 produced by P6.50, changing the shape of TMH6, when W6.48 moves from a χ1 of g+ to trans. It is likely that F3.36 is the other part of the GPR6 Toggle Switch, keeping W6.48 in a g+ χ1 in the inactive state. To test if F3.36/W6.48 is the GPR6 toggle switch [48], we will perform F3.36L/A, W6.48L/A mutations. We expect that mutations to smaller residues (L or A) will increase constitutive signaling of GPR6 as well as increase the Ki for the pyrazine analogs. F6.51 forms a stacking interaction with the pyrazine analogs docked here too. We expect that the F6.51A mutation will increase the Ki more than F6.51L mutation for these analogs.

**Site 3: TMH1-2-7.** Residue 2.60 is found to be a binding site residue in many Class A GPCRs, such as GPR55 [55,56]. This residue is a histidine in GPR6. In our docking studies, H2.60 is involved in an aromatic stacking interaction with core B of the pyrazine analogs. Similarly, W1.35 and Y7.36 also can form aromatic stacking with core B. To test this, the following mutants will be evaluated: H2.60V/A, W1.35V/A and Y7.36V/A. The H2.60V, W1.35V and Y7.36V mutations remove aromaticity, but retain some bulk, while H2.60A, W1.35A and Y7.36A remove both aromaticity and bulk. We expect that the valine mutants will have higher Ki’s and the A mutants will show even larger increases in Ki.

These mutation studies would help to test our hypotheses and refine our GPR6 model. As well, it will increase our structural knowledge for the next phase of the GPR6 project, in which scaffold hopping will be used to design new GPR6 ligands.

## 3. Materials and Methods

### 3.1. Amino Acid Numbering System

The Ballesteros–Weinstein numbering system for GPCR residues is used in this paper. This system is based on assigning the label, 0.50, to the most highly conserved amino acid in Class A GPCRs for each TMH [57]. This is preceded by the TMH number. In this system, for example, the most highly conserved residue in TMH1 is 1.50. In GPR6, this is N1.50. The residue before this in GPR6 would be labeled E1.49, and the one placed immediately after this would be labeled A1.51. Each TMH number can also contain the absolute sequence number in parentheses. Loop residues and N- and C-termini residues are designated with their absolute sequence number in parentheses only, such as the important E2 loop residue, R(220).

### 3.2. Receptor Model Development

A homology model of hGPR6 was constructed based upon the X-ray crystal structure of the Sphingosine-1-phosphate receptor 1 (S1P1) [PDB identifier: 3V2Y] [28], another class A GPCR. S1P1 was used as a template due to its 33% sequence homology (similarity) with GPR6 and because both share essential features such as (1) the absence of helix kinking proline residues in TMH2 (2.58 or 2.59) and TMH5 (5.50); (2) both possess an acidic residue E1.49 before the highly conserved N1.50 in TMH1 (most Class A GPCRs have a G1.49 here); and (3) the presence of an internal disulfide bridge in the EC2 loop. Neither the Lysophosphatidic acid receptor 1 (LPA1) [PDB identifier: 4Z35] [36] or CB1 [PDB ID: 5TGZ and 5U09] [58,59], the other closely related crystallized GPCRs, were used because the LPA1 shares less essential structural features than S1P1 and the CB1 structures suffer from crystal packing problems [60]. Before mutating, the sequence of GPR6 was aligned first with the sequences of other class A GPCRs such as Rhodopsin (Rho), CB1, CB2, δ-opioid receptor (DOR), β2-AR, LPA1 and S1P1 receptors using the highly conserved residues/motifs as alignment guides (N1.50, D2.50, R3.50, W4.50, Y5.58, CWXP in TMH6, and NPXXY in TMH7) (blue residues in Figure 1). Based on this alignment, the S1P1 model was mutated to GPR6 sequence. Some important sequence differences between S1P1 and GPR6 in TMHs 1,6 and 7 were identified and needed to be explored (red residues in Figure 1). These all involved the presence of additional helix bending residues (S/T, G, P): T1.44 in a SGT motif in TMH1, T6.43 in a GT motif that is near the CWXP motif in TMH6 and the presence of proline at position 7.41 at the top of TMH7 [31,32,38]. To explore the effect of these helix bending residues on helix shape, the Conformational Memories (CM) technique was used [29,30].

### 3.3. Conformational Memories Technique for Calculating TMH Conformations

Key sequence divergences between the template and GPR6 were explored using Conformational Memories [29,30]. Conformational Memories is a simulated annealing/Monte Carlo (MC) conformational analysis method that can be used to explore the sequence dictated range of conformations available at biological temperatures (310 K) for a TMH. This technique has been previously validated in the development of GPCR models [61]. In this method, the conformational consequences of helix bending residues were extensively and effectively explored in two separate phases. In the exploratory phase, the starting temperature for the simulated annealing (SA) was 3000 K, with cooling to 310 K in 18 temperature steps. At each temperature, 50,000 MC steps were applied. With each step, two dihedral angles and one bond angle were varied in the range of ±180° for dihedrals and of ±8° for bond angles. Conformers were accepted or rejected using the Metropolis criterion [62]. In the biased sampling phase, CM explores only regions that were highly populated in the first phase. The starting temperature was 749.4 K, with cooling in nine temperature steps to 310 K. At each temperature, 50,000 MC steps were applied, and the Metropolis criterion was used to accept or reject conformers. All calculations were performed using a distance-dependent dielectric and the PARAM22 force field [63,64,65] without CHARM corrections [66].

### 3.4. Modeling Loops and N- and C-Termini Conformations

The following residues were generated and added to the GPR6 model; the N- terminus (L(44) to L(69),) the intra- and extra-cellular loops (IC1: A(102) to T(105), IC2: A(173) to T(181), IC3: H(255) to A(273), EC1: L(134) to S(137), EC2: G(206) to A(223), EC3: S(303) to P(307) and the initial portion of the C-terminus, called HX8 (Q(334) to C(347)). MODELLER 8.2 (copyright © 2020–2017 Andrej Sali) was used to refine loop conformations [67,68,69]. Prime Homology Modeling implemented in the Schrödinger package (Schrödinger 2019-3: Prime, Schrödinger, LLC, New York, NY, USA, 2019) was used to add the N- terminus [70]. The following structural features noticed from other lipid sensing receptors were taken into consideration when modeling and selecting loops. These include: an N-terminus that covers the EC region, helping to block the access of ligand from the extracellular space above and shielding the binding site from water [28,41,43]; a limited distance introduced between C(209) and C(216) in the EC2 loop to form a highly conserved disulfide bridge and specific residues in the EC2 loop that point down into the binding crevice [28,41]. The final loops were energy minimized to a gradient of 0.05 kJ/mol Å with 2500 maximum iterations using the generalized Born/surface area (GB/SA) continuum solvation model for water and the OPLS3e [71,72,73,74] field in Macromodel 10.7.014 (Schrödinger Release 2017-4: MacroModel, Schrödinger, LLC, New York, NY, USA, 2017), while the TMH region was frozen. HX8, a short amphipathic helical section of the C-terminus that lies parallel to the cell membrane, was minimized using the same protocol described above for the loops, while all other atoms in the receptor were frozen.

### 3.5. Receptor Minimization

The resultant model was energy minimized using the protocol previously reported by our lab [51]. The minimization protocol used the OPLS3e [71,72,73,74] all-atom force field in Macromodel (Schrödinger 2017-4: MacroModel, Schrödinger, LLC, New York, NY, USA, 2017). An extended Van der Waals cutoff (8.0 Å, updated every 10 steps), a 20.0 Å electrostatic cutoff, and a 4.0 Å hydrogen bond cutoff were used in each stage of the calculation.

### 3.6. Molecular Dynamics (MD)

The GPR6 model was oriented with respect to the membrane using the template, 3V2Y, from the Orientation of Proteins in Membranes database [52] and placed in a hydrated 1-palmitoyl-2-oleoyl-sn-glycero-3-phosphocholine (POPC) bilayer using the CHARMM-GUI server [75,76]. The resulting system contained 156 POPC molecules, 13,857 water molecules, and an ionic strength of 0.15 M NaCl. The resulting simulation cell contained 67,191 atoms and was 80.2 Å × 80.2 Å × 113.1 Å. All molecular dynamics runs employed the all atom additive CHARMM36m force field for proteins [77], and CHARMM36 for lipid and ions [63]. The system was energy minimized and relaxed using multistep schedule as described previously [76]. Subsequent to this relaxation, 100 ns of MD was performed. MD simulation was performed with the GPU version of AMBER 18 [78,79] in the semi-isotropic NPT ensemble using a Langevin thermostat (T = 310 K, using a friction coefficient of 1 ps^−1^) and a Monte Carlo barostat (P = 1 bar) as implemented in AMBER 18. Long range electrostatics were treated using PME (particle mesh ewald) and the Van der Waal force switching method was applied starting at 10 Å with a cutoff of 12 Å. High-frequency bonds to hydrogen were constrained using the SHAKE method, allowing the use of a 2-fs integration time step. Finally, a sodium ion was positioned in the putative sodium ion binding site. This site includes the polar residues, N1.50, D2.50, S3.39, N7.49, and Y7.53, and directly coordinates a sodium ion to stabilize the inactive state of the receptor [80,81,82,83]. The RMSD plot for the TMH bundle versus time was ere used to analyze the stability of the bundle in the bilayer.

### 3.7. Conformer Analysis of Pyrazine Analogs

Complete conformational analyses of the selected analogs were performed using ab initio Hartree−Fock calculations at the 6-31G* level, within the Spartan molecular modeling program (Wave function, Inc., Irvine, CA). Hartree Fock 6- 31G* 3-fold or 2-fold rotation conformer searches were performed for all the rotatable bonds (For compounds **A-415**, **B-582**, **C-2, D-33**, **G-133, H-33,** and **I-247** these were: C2-N1′, N1′-C2′, C3-N1″, C4″/N4″-C7″/O7″, and C7″/O7″-C8″ bonds, with the addition of C5-C1‴, and C1‴-N2‴ bonds in compound **B-582**; C7-C1‴, C1‴-C2‴and C2‴-O3‴ bonds in compound **C-2**, C6-C1‴; and C1‴-O2‴ bonds in compound **D-33**. For compound **E-5** these were: C2-N1′, N1′-C2′, C2′-C3′, C3-N1″, C4‘’-O7″, O7″-C8″, and C6-S1‴ bonds). In each conformer search, local energy minima were identified by rotation of a subject torsion angle through 360° in 120° increments (3-fold search) or in 180° increments (2-fold search), followed by HF 6-31G* energy minimization of each rotamer generated (see Supplementary Materials, Figure S3). 

To calculate the difference in energy between the global minimum energy conformer of each compound and its final docked conformation, the global minimum conformer as determined by Hartree Fock 6-31 G* was first relaxed in the OPLS3e forcefield to a gradient of 0.01 kcal/((mol) (Å^2^)). The OPLS3e based single point energy of each ligand’s docked conformation, extracted from each of the minimized ligand/receptor complexes was calculated. Then, the energy difference for each compound was calculated by subtracting the potential energy of the global minimum conformer from the docked conformer (see Supplementary Materials for the conformational cost for each compound). Each calculation was performed in Macromodel with a Generalized Born Solvent Accessibility Model, or GBSA, for water and a constant dielectric of 1.

### 3.8. Electrostatic Potential Map Calculation

The electrostatic potential density surface of the global minimum conformation of the ligands was calculated using Spartan’08 (Wave function, Inc., Irvine, CA, USA). The electrostatic potential energy was calculated using the Hartree−Fock method at the 6-31G* energy level of theory and was mapped on the 0.002 isodensity surface of each ligand. The electron-rich regions are colored red and the electron-poor regions are colored blue (see Supplementary Materials, Figure S2).

### 3.9. Docking of Ligands

The global minimum conformer of **A-415**, **B-582**, **C-2**, **D-33**, **E-5, G-133, H-33,** and **I-247** was used as input for manual docking into the receptor model. These analogs were docked using interactive graphics in the binding site of the GPR6 R model. The receptor/ligand complex was then minimized in Prime (Schrödinger 2019-3: Prime, Schrödinger, LLC, New York, NY, USA, 2019) using the OPLS3e all atom force field with the implicit slab membrane model [71], Variable-Dielectric Solvation Generalized Born Model (VSGB) for water [84], and a constant dielectric of 80 for outside the inner slab. The Orientation of Proteins in Membranes, or OPM, was used to provide membrane information to orient the receptor/ligand complexes during the minimization and set the depth of the membrane slab at 31.8 Å [52]. Cartesian constraints were applied to the TMH backbones with the coefficient of the harmonic constraint potential set to 1 kcal mol^−1^ Å^−2^ and a flat bottom well of 0.5 Å. Glide score of the final minimized receptor/ligand complexes were calculated using standard precision (Glide SP) with a box size of 20 Å^3^ encompassing the binding crevice, score in place docking method, dielectric constant set at 2, halogens treated as acceptors and aromatic hydrogens treated as donors [53].

### 3.10. Calculation of Ligand/Receptor Interaction Energy

Assessment of Pairwise Interaction and Total Interaction Energies between each bound ligand and residue in the GPR6 R complex were calculated using Macromodel 9.3 (Schrödinger 2019-3: MacroModel, Schrödinger, LLC, New York, NY, USA, 2019), as described previously [85]. Specifically, after defining the atoms of the ligand as one group (group 1) and the atoms corresponding to a residue that lines the binding site in the final ligand-GPR6 R complex as another group (group 2), Macromodel was used to output the pairwise interaction energy (Coulombic and van der Waals) for a given pair between the ligand and each residue within a 5 Å radius. The energies were calculated with an extended cutoff (non-bonded: 8.0 Å, electrostatic: 20.0 Å, hydrogen bonding: 4.0 Å) and a distance dependent dielectric of 2.

## 4. Conclusions and Future Perspectives

GPR6 has emerged as a promising therapeutic target for the treatment of neurological diseases such as Parkinson´s disease and other dyskinesia syndromes. However, the lack of availability of ligands identified so far is delaying the exploitation and understanding of this orphan GPCR. In an effort to deepen our understanding of the GPR6 structure, we have constructed an inactive state model to rationalize the key molecular features for the activity of reported inverse agonists.

An analysis of patented molecules and subsequent docking studies of selected compounds in our model led us to identify a putative orthosteric binding pocket for these ligands. Our docking experiments suggest that crucial interaction sites for these inverse agonists involve R(220), from the EC2 loop, toggle switch residues F3.36, W6.48, F6.51 and the aromatic residues in the TMH1-2-7 region: W1.35, H2.60, F2.61 and/or Y7.36.

The elucidation of the crucial interacting sites for these inverse agonists can open new avenues for the optimization of these ligands. In addition, mutagenesis and consequent receptor refinement will be performed in the near future to test our hypothesis.

## Figures and Tables

**Table 1:** ProKink analysis [36] results for the chosen TMH conformers for inclusion in GPR6 Model.

Helix	Hinge Residue	Bend Angle (deg)	Wobble Angle (deg)	Face Shift (deg)
TMH1	T1.44	13.0	172.4	3.0
TMH6	T6.43	9.9	113.0	5.8
TMH7	P7.41	12.7	−20.1	31.9

## Supplementary Materials

The following are available online. Figure S1: Human sequence alignments of S1PR1 and GPR6 receptors. Figure S2: Molecular electrostatic potential maps of conformations of docked ligands. Figure S3: Atom numbering description of GPR6 modulators—Pyrazine analogs used for conformational analysis. Figure S4: **G-133**/GPR6 R complex. Figure S5: **H-33**/GPR6 R complex. Figure S6: **I-247**/GPR6 R complex. Table S1: Distance and angle measurements for interactions found in ligand/GPR6 R complexes Table S2: Compound **A-415**/GPR6 R complex interaction energy decomposition. Table S3: Compound **B-582**/GPR6 R complex interaction energy decomposition. Table S4: Compound **C-2**/GPR6 R complex interaction energy decomposition. Table S5: Compound **D-33**/GPR6 R complex interaction energy decomposition. Table S6: Compound **E-5**/GPR6 R complex interaction energy decomposition. Table S7: Compound **G-133**/GPR6 R complex interaction energy decomposition. Table S8: Compound **H-33**/GPR6 R complex interaction energy decomposition. Table S9: Compound **I-247**/GPR6 R complex interaction energy decomposition.

## Abbreviations

GPCR	G-Protein Coupled Receptor
cAMP	cyclic AMP
CHO	Chinese hamster ovary
S1P1	Sphingosine-1-phosphate receptor 1
CM	Conformational Memories
TMH	Transmembrane helix
EC	Extracellular
IC	Intracellular
CB1	Cannabinoid receptor type 1
MD	Molecular dynamics
RMSD	root-mean-square deviation
SAR	Structure-Activity Relationship
MBER	Assisted Model Building with Energy Refinement
