# Supplementary material for: GPR6 Structural Insights: Homology Model Construction and Docking Studies
Source: Molecules. 2020 Feb 7;25(3):725. doi: 10.3390/molecules25030725 (PMC7037797; doi:10.3390/molecules25030725)
Supplement: Supplementary file 1 [file molecules-25-00725-s001.pdf]

molecules

Supplementary Materials

GPR6 Structural Insights: Homology Model Construction and Docking Studies

Israa H. Isawi [1], Paula Morales [2], Noori Sotudeh [3], Dow P. Hurst [1], Diane L. Lynch [1], and Patricia H. Reggio [1,*]

[1] Department of Chemistry and Biochemistry, University of North Carolina at Greensboro, Greensboro, NC 27412, USA; ihisawi@uncg.edu (I.H.I.), dphurst@uncg.edu (D.P.H.); dllynch@uncg.edu (D.L.L.).
[2] Instituto de Química Medica (IQM-CSIC), C/Juan de la Cierva 3, 28006 Madrid, Spain; paula.morales@iqm.csic.es
[3] Department of Physiology and Biophysics, The State University of New York at Buffalo, Buffalo, NY, 14260, USA; nouroddi@buffalo.edu
* Correspondence: phreggio@uncg.edu

Content

Figure S1: Human sequence alignments of S1PR1 and GPR6 receptors.

Figure S2: Molecular electrostatic potential maps of conformations of docked ligands.

Figure S3: Atom numbering description of GPR6 modulators - Pyrazine analogs used for conformational analysis.

Figure S4: G-133/GPR6 R complex.

Figure S5: H-33/GPR6 R complex.

Figure S6: I-247/GPR6 R complex.

Table S1: Distance and angle measurements for interactions found in ligand/GPR6 R complexes.

Table S2: Compound **A-415**/GPR6 R complex interaction energy decomposition

Table S3: Compound **B-582**/GPR6 R complex interaction energy decomposition.

Table S4: Compound **C-2**/GPR6 R complex interaction energy decomposition.

Table S5: Compound **D-33**/GPR6 R complex interaction energy decomposition.

Table S6: Compound **E-5**/GPR6 R complex interaction energy decomposition.

Table S7: Compound **G-133**/GPR6 R complex interaction energy decomposition.

Table S8: Compound **H-33**/GPR6 R complex interaction energy decomposition.

Table S9: Compound **I-247**/GPR6 R complex interaction energy decomposition.

Figure S1: Human sequence alignments of S1PR1 and GPR6 receptors. IC: Intracellular Loop, EC: Extracellular Loop. Color code: yellow and green: highly conserved residues, red: prolines, pink: cysteines in an internal disulfide bridge, grey: transmembrane residues.

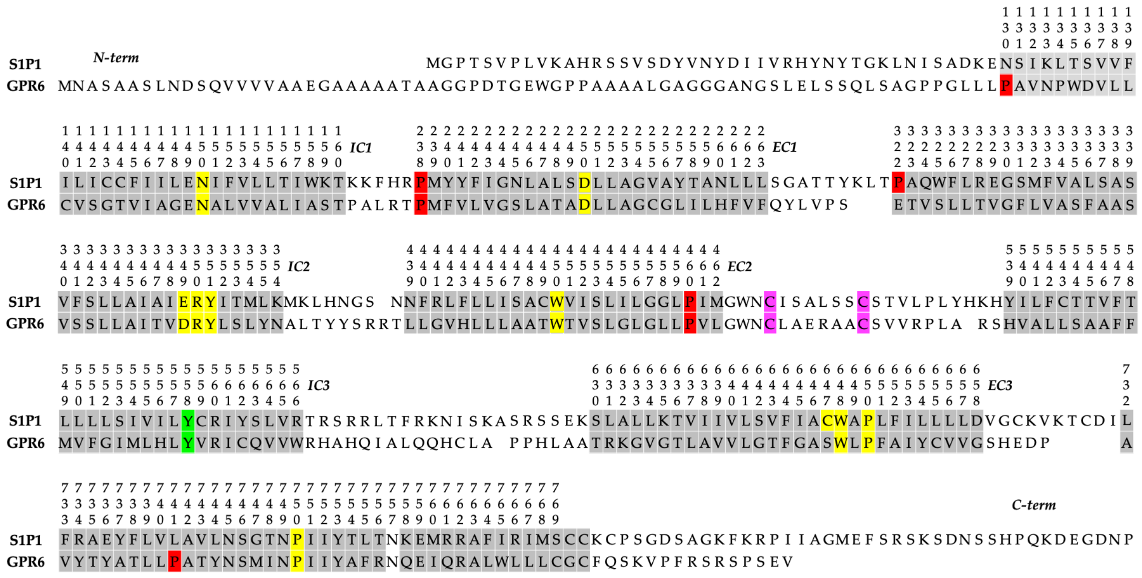

Figure S2: Molecular electrostatic potential maps of the global minimum conformations of docked ligands. The electrostatic potential scale (in kJ/mol) is provided as a color scale. This scale is from blue (most electropositive) to red (most electronegative). The numbers next to the alphabetic letter for each compound come from Envoy Therapeutics, Inc. and Takeda Pharmaceutical Company patents.

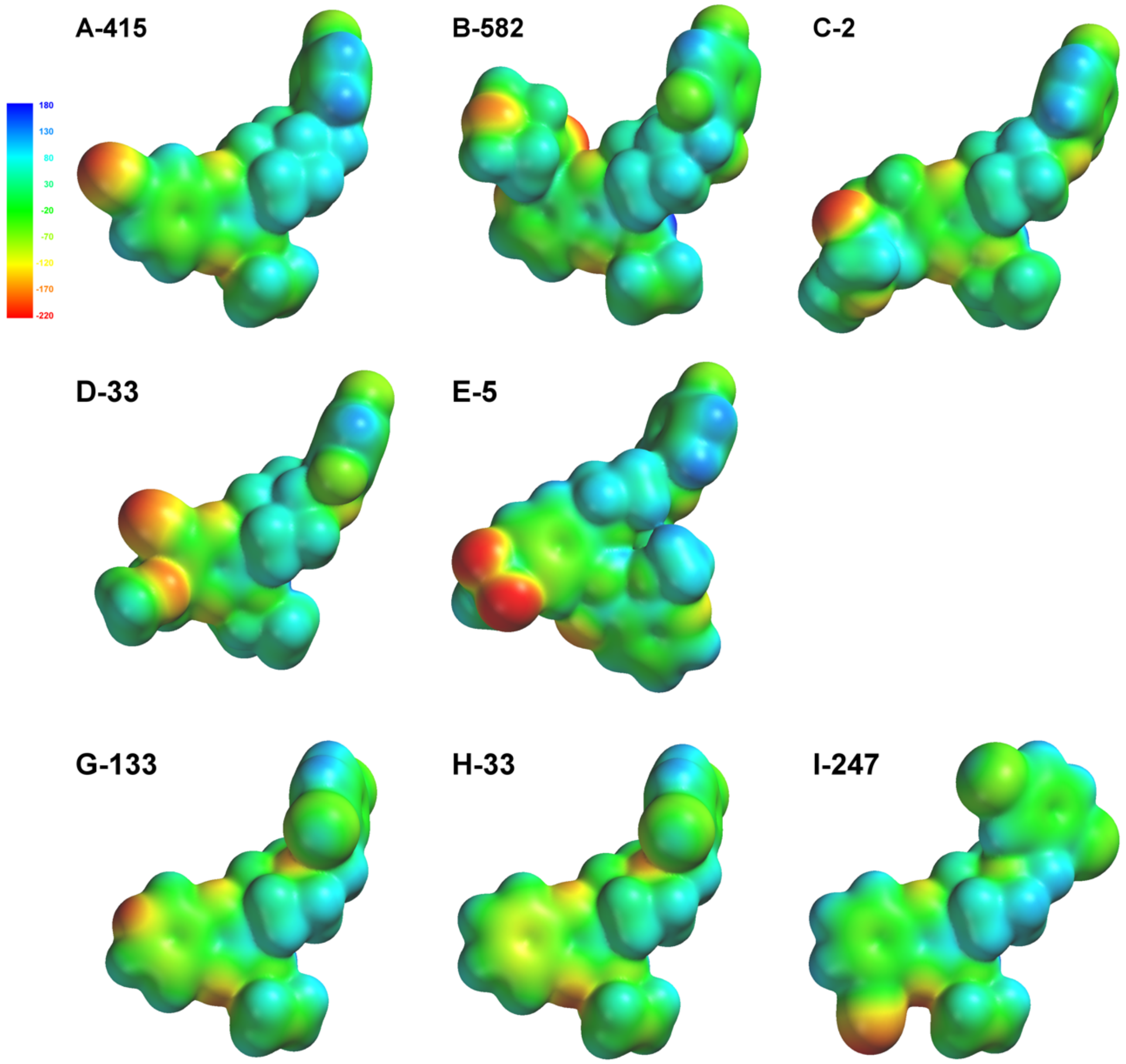

Figure S3: Atom numbering description of GPR6 modulators - Pyrazine analogs used for conformational analysis. Conformer searches were performed for all red rotatable bonds.

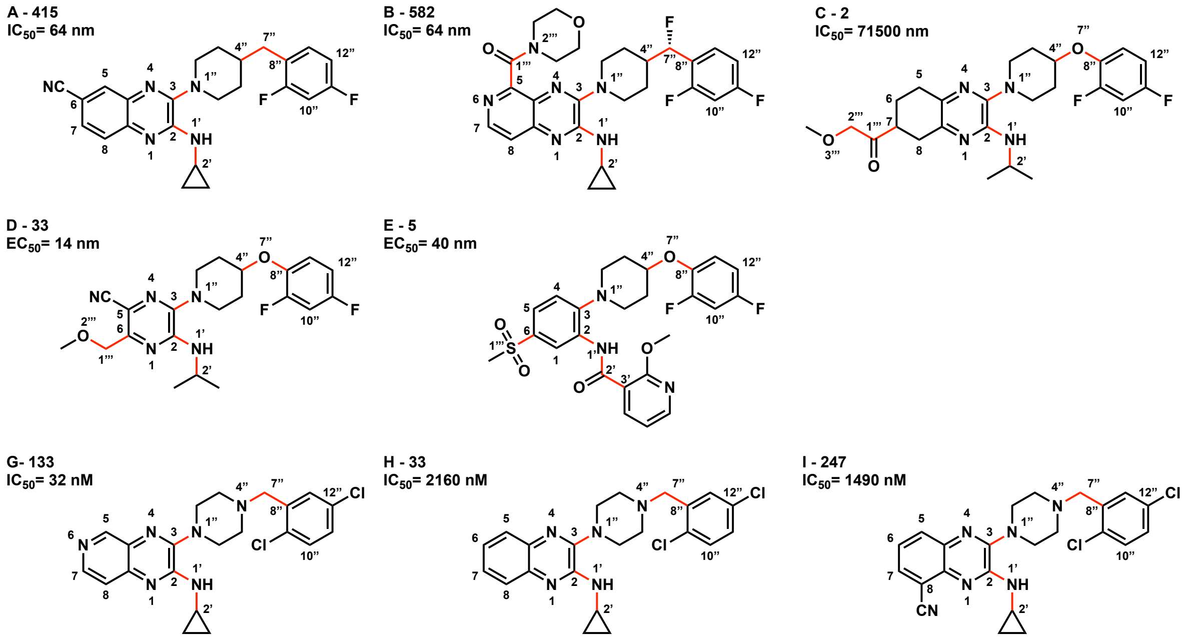

Table S1: Distance and angle measurements for interactions found in ligand/GPR6 R complexes.

Interaction Type	Residue/Atom		Measurements	Ligands							
				A-415	B-582	C-2	D-33	E-5	G-133	H-33	I-247
Aromatic Stacking	W1.35(75)		Distance (Å)	5.19	-	6.43	5.74	-	-	-	5.59
			Angle (deg)	60	-	52	56	-	-	-	76
Aromatic Stacking	H2.60(128)		Distance (Å)	6.88	5.78	5.41	5.71	5.40	-	-	5.55
			Angle (deg)	77	47	87	85	76	-	-	57
Aromatic Stacking	F2.61(129)		Distance (Å)	-	-	-	-	6.46	-	-	-
			Angle (deg)	-	-	-	-	78	-	-	-
Aromatic Stacking	F3.36(152)		Distance (Å)	5.77	5.77	-	-	-	5.50	5.38	-
			Angle (deg)	78	65	-	-	-	84	87	-
Hydrogen Bond	R(220)	NE–X	Distance (Å)	3.13	2.91	-	3.14	2.86	-	-	-
		NE–H- -X	Angle (deg)	149	145	-	148	175	-	-	-
		NH1–X	Distance (Å)	-	-	-	-	-	3.11	-	-
		NH1–H- -X	Angle (deg)	-	-	-	-	-	144	-	-
		NH2–X	Distance (Å)	3.00	2.78	2.91	3.04	2.70	3.00	-	-
		NH2–H- -X	Angle (deg)	153	147	147	151	166	147	-	-
Aromatic Stacking	W6.48(292)		Distance (Å)	4.93	-	-	5.90	5.43	5.32	5.34	-
			Angle (deg)	44	-	-	35	79	58	61	-
Aromatic Stacking	F6.51(295)		Distance (Å)	-	5.42	5.59	5.29	5.85	5.34	5.27	6.10
			Angle (deg)	-	78	54	72	72	86	85	76
Aromatic Stacking	Y7.36(312)		Distance (Å)	5.96	-	-	-	-	5.21	-	-
			Angle (deg)	60	-	-	-	-	50	-	-

X: represent ligand’s heteroatom that forms the hydrogen bond interaction with R220.

48 **Table S2:** Compound **A-415**/GPR6 R complex interaction energy decomposition.

Compound **A-415**			
Residue	Electrostatic (kcal/mol)	Van der Waals (kcal/mol)	Total Energy (kcal/mol)
L3.32(148)	-0.06	-7.01	-7.07
R(220)	-6.22	-0.06	-6.28
L7.39(315)	0.05	-4.75	-4.7
L2.57(125)	0.22	-3.87	-3.65
T7.43(319)	-0.22	-3.05	-3.27
W6.48(292)	-0.02	-2.39	-2.42
V3.33(149)	-0.13	-2.18	-2.31
L(60)	-0.1	-2.09	-2.19
Q(132)	0.18	-2.35	-2.17
F6.51(295)	-0.17	-1.84	-2.01
Y7.36(312)	-0.48	-1.44	-1.92
W1.35(75)	0.16	-1.92	-1.77
A2.53(121)	0.13	-1.83	-1.7
F3.36(152)	0.09	-1.78	-1.69
A7.42(318)	0.00	-1.66	-1.66
L(56)	-0.04	-1.42	-1.46
V(135)	-0.08	-1.11	-1.19
G2.54(122)	-0.06	-0.81	-0.87
S7.46(322)	0.03	-0.81	-0.78
L1.39(79)	0.02	-0.75	-0.73
H2.60(128)	-0.09	-0.5	-0.59
V3.29(145)	-0.01	-0.51	-0.51
D2.50(118)	-0.24	-0.19	-0.43
F2.61(129)	-0.05	-0.35	-0.41
T3.28(144)	-0.01	-0.28	-0.3
L7.40(316)	-0.04	-0.23	-0.26
S(57)	0.01	-0.26	-0.25
S3.35(151)	0.06	-0.3	-0.24
L5.43(230)	-0.06	-0.16	-0.22
I1.46(86)	0.02	-0.21	-0.19
Interaction Energy			-53.21
Conformational Energy			1.6
Total Energy			-51.71

Glide Score			-10.10

Table S3: Compound **B-582**/GPR6 R complex interaction energy decomposition.

Compound **B-582**			
Residue	Electrostatic (kcal/mol)	Van der Waals (kcal/mol)	Total Energy (kcal/mol)
R(220)	-8.85	-2.66	-11.51
L7.39(315)	0.06	-6.25	-6.19
L3.32(148)	-0.08	-5.57	-5.64
W6.48(292)	-0.02	-2.72	-2.75
V3.33(149)	-0.12	-2.40	-2.52
C6.55(299)	-0.60	-1.72	-2.32
V(219)	0.36	-2.67	-2.31
F3.36(152)	-0.17	-2.14	-2.30
T7.43(319)	-0.24	-1.97	-2.21
F6.51(295)	0.21	-2.40	-2.20
H2.60(128)	-0.44	-1.38	-1.82
L2.57(125)	0.00	-1.58	-1.58
Y7.36(312)	-0.21	-1.17	-1.38
V(135)	0.00	-1.26	-1.27
L(56)	0.06	-1.18	-1.12
V3.29(145)	0.01	-1.12	-1.11
A7.42(318)	0.05	-1.09	-1.05
S(57)	-0.04	-0.93	-0.97
T7.35(311)	-0.07	-0.68	-0.75
A2.53(121)	0.08	-0.65	-0.57
S(53)	0.12	-0.65	-0.54
W1.35(75)	0.00	-0.42	-0.42
S(217)	-0.03	-0.36	-0.39
T3.28(144)	0.03	-0.36	-0.33
V(218)	0.11	-0.39	-0.28
L5.43(230)	-0.01	-0.19	-0.20
V5.40(227)	-0.01	-0.10	-0.12
G2.54(122)	-0.01	-0.10	-0.11
L(54)	0.01	-0.11	-0.10
S7.46(322)	0.01	-0.09	-0.08
S3.35(151)	0.06	-0.12	-0.06
Interaction Energy			-54.17
Conformational Energy			2.5
Total Energy			-51.67

Glide Score			-8.72

Table S4: Compound **C-2** /GPR6 R complex interaction energy decomposition.

Compound **C-2**			
Residue	Electrostatic (kcal/mol)	Van der Waals (kcal/mol)	Total Energy (kcal/mol)
L3.32(148)	-0.33	-5.96	-6.28
R(220)	-5.98	-0.01	-5.99
F6.51(295)	0.21	-4.45	-4.24
L7.39(315)	-0.02	-3.9	-3.92
L2.57(125)	0.02	-3.76	-3.73
W6.48(292)	0.2	-3.46	-3.26
F3.36(152)	0.27	-3.27	-3.00
H2.60(128)	-0.57	-1.92	-2.49
T7.43(319)	-0.18	-1.91	-2.1
V3.29(145)	0.07	-2.00	-1.93
V3.33(149)	0.10	-1.92	-1.82
A2.53(121)	0.03	-1.62	-1.59
T3.28(144)	-0.11	-1.00	-1.11
V(135)	-0.04	-1.02	-1.06
C6.55(299)	0.00	-1.04	-1.04
A6.52(296)	0.06	-1.05	-0.99
L5.43(230)	-0.04	-0.82	-0.86
W1.35(75)	0.01	-0.8	-0.80
A7.42(318)	-0.01	-0.67	-0.69
F5.47(234)	0.06	-0.72	-0.67
S3.35(151)	0.07	-0.5	-0.44
Y7.36(312)	-0.49	-1.16	-0.64
S3.25(141)	-0.01	-0.29	-0.3
S5.44(231)	-0.01	-0.25	-0.25
G2.54(122)	0.02	-0.22	-0.2
Q(132)	0.03	-0.22	-0.2
S7.46(322)	0.02	-0.11	-0.09
L(60)	0.02	-0.09	-0.08
Interaction Energy			-49.77
Conformational Energy			2.6
Total Energy			-47.17
Glide score			-7.69

Table S5: Compound **D-33** /GPR6 R complex interaction energy decomposition.

Compound **D-33**			
Residue	Electrostatic (kcal/mol)	Van der Waals (kcal/mol)	Total Energy (kcal/mol)
R(220)	-6.54	-0.84	-7.38
L3.32(148)	-0.23	-5.35	-5.59
L7.39(315)	-0.1	-4.92	-5.01
W6.48(292)	-0.11	-4.45	-4.56
F3.36(152)	-0.2	-4.22	-4.41
L2.57(125)	0.01	-3.43	-3.42
V3.33(149)	-0.09	-2.91	-3.00
F6.51(295)	-0.25	-2.63	-2.88
T7.43(319)	-0.35	-1.97	-2.32
Q(132)	-0.06	-1.77	-1.84
W1.35(75)	-0.14	-1.58	-1.72
H2.60(128)	-0.23	-1.48	-1.71
V3.29(145)	-0.01	-1.41	-1.42
Y7.36(312)	0.44	-1.67	-1.23
A2.53(121)	0.09	-1.31	-1.22
L(60)	-0.02	-0.96	-0.99
V(135)	-0.05	-0.78	-0.83
S3.35(151)	0.13	-0.75	-0.62
A7.42(318)	-0.04	-0.44	-0.47
L7.40(316)	-0.08	-0.34	-0.42
T3.28(144)	0.05	-0.43	-0.38
L5.43(230)	-0.07	-0.26	-0.33
P5.47(234)	-0.03	-0.19	-0.21
G2.54(122)	-0.01	-0.16	-0.17
S7.46(322)	0.03	-0.13	-0.1
A6.52(296)	0.05	-0.09	-0.03
S3.39(155)	0.06	-0.05	0.01
Interaction Energy			-52.26
Conformational Energy			1.9
Total Energy			-50.36
Glide Score			-8.71

Table S6: Compound **E-5**/GPR6 R complex interaction energy decomposition.

Compound **E-5**			
Residue	Electrostatic (kcal/mol)	Van der Waals (kcal/mol)	Total Energy (kcal/mol)
R(220)	-11.14	-0.15	-11.3
L3.32(148)	-0.59	-5.77	-6.36
L7.39(315)	-0.12	-6.06	-6.18
L2.57(125)	-0.15	-4.04	-4.19
V3.29(145)	0.33	-4.03	-3.71
H2.60(128)	-0.08	-2.97	-3.04
F6.51(295)	-0.22	-2.48	-2.7
F2.61(129)	-0.18	-2.39	-2.57
W6.48(292)	-0.06	-2.47	-2.53
V3.33(149)	0.16	-2.68	-2.52
Y7.36(312)	0.07	-2.55	-2.48
Q(132)	-0.4	-1.47	-1.87
A7.42(318)	-0.28	-1.52	-1.8
T7.43(319)	-0.07	-1.61	-1.68
V(135)	-0.04	-1.27	-1.31
F3.36(152)	0.11	-1.35	-1.24
W1.35(75)	-0.01	-1.14	-1.15
A2.53(121)	0.16	-1.11	-0.95
T3.28(144)	0.05	-0.97	-0.91
V(219)	-0.20	-0.5	-0.7
L1.39(79)	0.01	-0.46	-0.45
L(60)	0.05	-0.49	-0.43
C6.55(299)	0.35	-0.62	-0.27
L7.40(316)	-0.05	-0.21	-0.26
S3.35(151)	0.19	-0.41	-0.22
L5.43(230)	-0.05	-0.14	-0.18
G3.30(146)	0.01	-0.15	-0.14
S7.46(322)	0.09	-0.23	-0.13
G2.56(124)	0.01	-0.14	-0.13
L(56)	0.01	-0.09	-0.08
L(222)	0.00	-0.06	-0.06
P(221)	0.03	-0.09	-0.06
Interaction Energy			-61.62
Conformational Energy			2.4

Total Energy			-59.22
Glide Score			-10.38

Figure S4: G-133/GPR6 R complex. The view from the lipid bilayer with the EC portions of TMH6-7 removed for clarity.

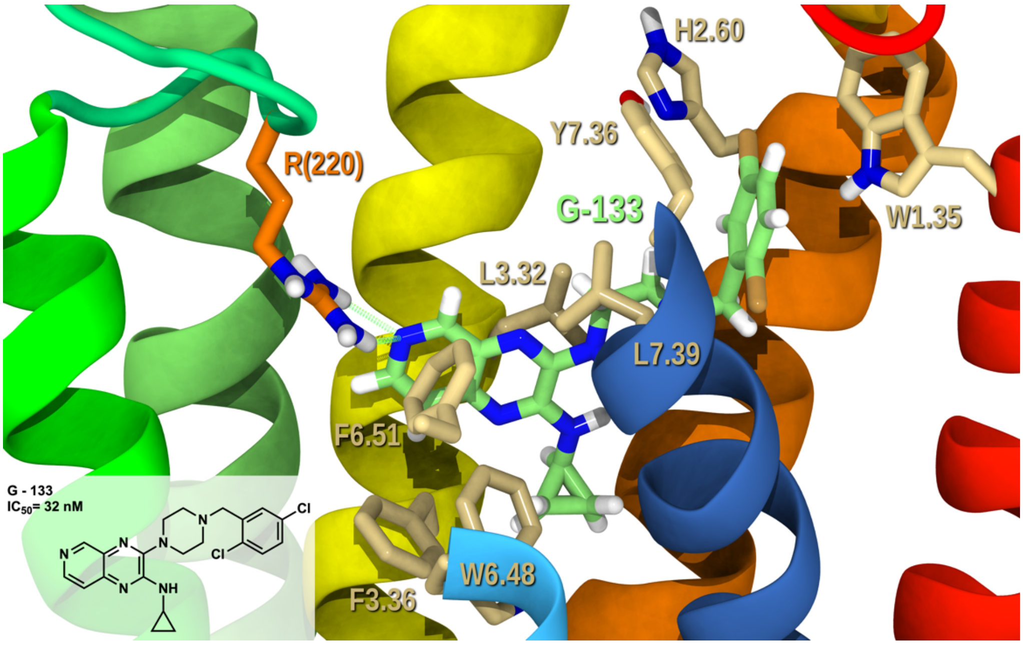

Figure S5: H-33/GPR6 R complex. The view from the lipid bilayer with the EC portions of TMH6-7 removed for clarity.

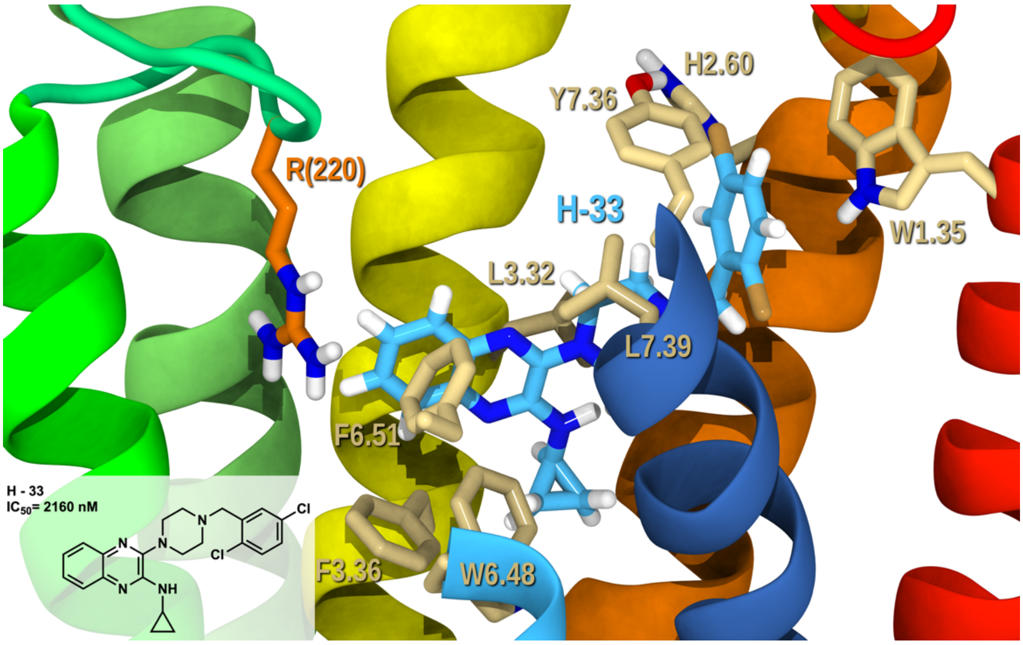

Figure S6: I-247/GPR6 R complex. The view from the lipid bilayer with the EC portions of TMH6-7 removed for clarity.

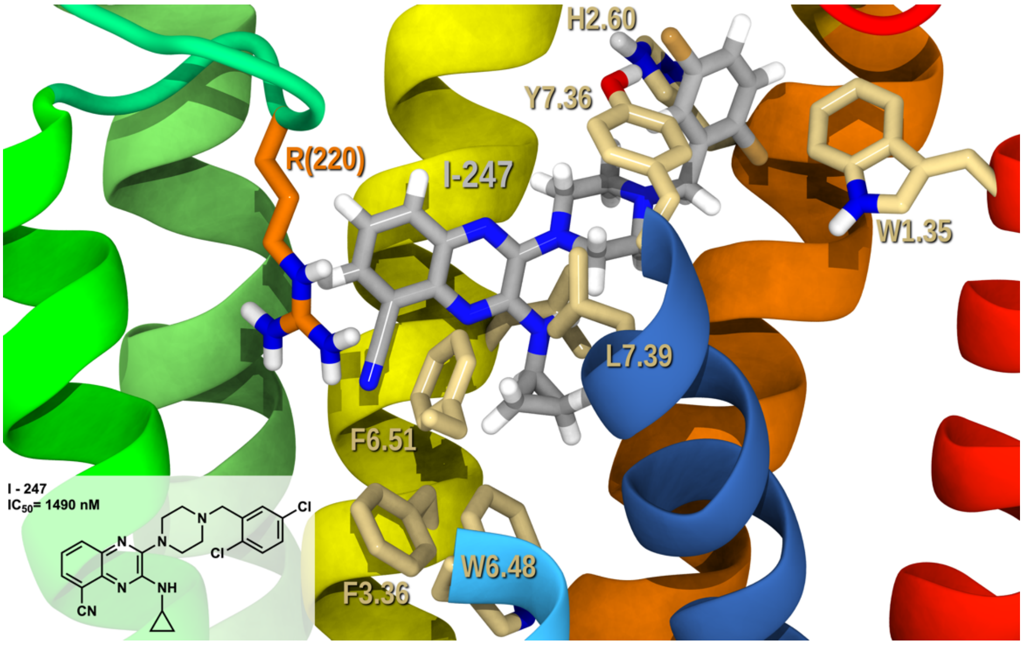

Table S7: Compound **G-133**/GPR6 R complex interaction energy decomposition.

Compound **G-133**			
Residue	Electrostatic (kcal/mol)	Van der Waals (kcal/mol)	Total Energy (kcal/mol)
L3.32(148)	0.05	-8.00	-7.95
L7.39(315)	-0.46	-7.14	-7.60
F3.36(152)	0.15	-3.45	-3.30
L2.57(125)	0.19	-3.43	-3.24
R(220)	-2.65	-0.31	-2.96
Y7.36(312)	-0.01	-2.85	-2.86
W6.48(292)	-0.07	-2.73	-2.80
V3.33(149)	0.03	-2.63	-2.60
W1.35(75)	-0.20	-2.13	-2.33
A7.42(318)	-0.03	-2.15	-2.18
T7.43(319)	-0.77	-1.37	-2.14
L1.39(79)	-0.01	-2.09	-2.10
F6.51(295)	-0.07	-1.58	-1.64
L(60)	0.02	-1.47	-1.45
L7.40(316)	0.08	-1.40	-1.32
S3.35(151)	-0.20	-0.79	-0.99
A2.53(121)	0.11	-0.88	-0.78
D2.50(118)	-0.30	-0.44	-0.74
V3.29(145)	0.06	-0.64	-0.57
V(135)	-0.03	-0.52	-0.55
S7.46(322)	-0.04	-0.40	-0.45
F2.61(129)	0.02	-0.37	-0.35
Q(132)	0.16	-0.40	-0.24
G2.54(122)	-0.04	-0.20	-0.24
A2.49(117)	-0.07	-0.15	-0.23
N7.45(321)	0.02	-0.18	-0.16
A3.37(153)	0.00	-0.09	-0.09
S3.39(155)	0.07	-0.13	-0.06
P(64)	0.01	-0.06	-0.05
Interaction Energy			-51.92
Conformational Energy			1.4
Total Energy			-50.52
Glide Score			-9.40

Table S8: Compound **H-33**/GPR6 R complex interaction energy decomposition.

Compound **H-33**			
Residue	Electrostatic (kcal/mol)	Van der Waals (kcal/mol)	Total Energy (kcal/mol)
L7.39(315)	-0.43	-7.49	-7.92
L3.32(148)	0.03	-7.22	-7.19
F3.36(152)	0.08	-3.48	-3.40
W6.48(292)	-0.12	-2.79	-2.91
L2.57(125)	0.12	-2.96	-2.84
W1.35(75)	-0.24	-2.45	-2.69
V3.33(149)	0.03	-2.61	-2.58
Y7.36(312)	0.14	-2.67	-2.53
A7.42(318)	-0.04	-2.05	-2.09
T7.43(319)	-0.75	-1.32	-2.08
F6.51(295)	-0.09	-1.37	-1.46
L7.40(316)	0.10	-1.52	-1.42
L1.39(79)	0.00	-1.33	-1.34
S3.35(151)	-0.18	-0.80	-0.99
H2.60(128)	-0.14	-0.85	-0.98
L(60)	-0.01	-0.96	-0.97
R(220)	0.65	-1.60	-0.95
A2.53(121)	0.07	-0.96	-0.89
D2.50(118)	-0.20	-0.36	-0.56
V3.29(145)	0.05	-0.57	-0.52
V(135)	-0.02	-0.32	-0.34
S7.46(322)	-0.01	-0.26	-0.27
A2.49(117)	-0.06	-0.14	-0.20
T3.28(144)	0.01	-0.19	-0.18
G2.54(122)	-0.03	-0.14	-0.17
N7.45(321)	0.01	-0.15	-0.14
A3.37(153)	0.00	-0.09	-0.09
S3.39(155)	0.06	-0.13	-0.07
P(64)	0.01	-0.06	-0.04
Interaction Energy			-47.82
Conformational Energy			2.2
Total Energy			-45.52
Glide Score			-9.90

Table S9: Compound **I-247**/GPR6 R complex interaction energy decomposition.

Compound **I-247**			
Residue	Electrostatic (kcal/mol)	Van der Waals (kcal/mol)	Total Energy (kcal/mol)
L3.32(148)	0.16	-5.99	-5.83
L7.39(315)	-0.03	-4.46	-4.49
R(220)	-0.67	-3.64	-4.31
V3.29(145)	0.06	-3.89	-3.83
H2.60(128)	-0.19	-3.21	-3.40
F6.51(295)	-0.19	-2.89	-3.08
Y7.36(312)	-0.73	-1.91	-2.65
V3.33(149)	0.02	-2.57	-2.55
L2.57(125)	0.42	-2.65	-2.24
Q(132)	-0.43	-1.48	-1.90
F3.36(152)	-0.22	-1.65	-1.87
V(135)	0.10	-1.94	-1.84
L(60)	-0.02	-1.81	-1.83
W1.35(75)	0.00	-1.67	-1.67
F2.61(129)	-0.04	-1.38	-1.42
T3.28(144)	0.08	-0.80	-0.71
A7.42(318)	0.00	-0.71	-0.70
W6.48(292)	0.07	-0.57	-0.50
T7.43(319)	-0.09	-0.38	-0.47
P(221)	0.13	-0.50	-0.38
L(56)	0.04	-0.40	-0.37
L1.39(79)	0.00	-0.31	-0.31
S3.25(141)	-0.05	-0.26	-0.31
S(57)	-0.01	-0.25	-0.26
V(219)	-0.01	-0.20	-0.21
A2.53(121)	-0.01	-0.18	-0.20
Interaction Energy			-47.35
Conformational Energy			1.7
Total Energy			-45.65
Glide Score			-8.16
